# A DFT Study on Deactivation of Triplet Excited State Riboflavin by Polyphenols

**DOI:** 10.3390/ijms9101908

**Published:** 2008-10-08

**Authors:** Hong-Fang Ji, Liang Shen

**Affiliations:** Shandong Provincial Research Center for Bioinformatic Engineering and Technique, Center for Advanced Study, Shandong University of Technology, Zibo 255049, P.R. China

**Keywords:** Riboflavin, polyphenols, triplet excited state, deactivation, density functional theory

## Abstract

The deactivation of triplet excited state riboflavin by polyphenols, *e.g.* rutin and catechin, was studied on the basis of density functional theory calculations. The results show that the H-atom transfer pathway is more feasible on thermodynamic grounds in comparison with the direct energy transfer or direct electron transfer pathways involved in the triplet excited state riboflavin deactivation by rutin/catechin. The findings are helpful to understand the protective effect of polyphenols against the riboflavin induced photosensitizing damage.

## 1. Introduction

**Figure 1.**
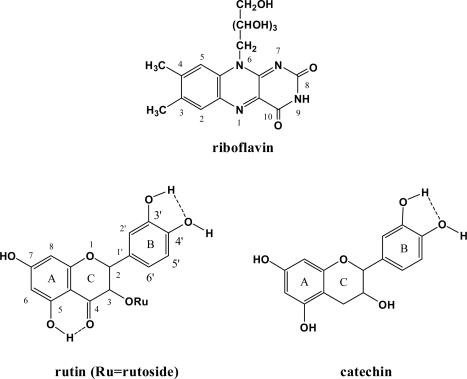
Molecular structures of riboflavin, rutin and catechin.

Numerous endogenous photosensitizers, among which riboflavin has attracted much attention, can photogenerate various reactive oxygen species (ROS, *e.g.* ^1^O_2_ and O_2_^˙−^) [1, 2]. It has been reported that riboflavin is an efficient ROS-generator [1, 3, 4] and can cause photosensitizing DNA damage [5, 6]. Polyphenolic compounds, *e.g.* rutin and catechin (Figure 1), are ideal antioxidants with strong free radical-scavenging ability. Recently, it was reported that rutin and catechin play dual roles in protecting from the photosensitizing damage caused by riboflavin, that is, as ROS scavengers and triplet excited (T_1_) state riboflavin quenchers [7]. The free radical-scavenging mechanisms of rutin and catechin have been investigated before [8], however, more effort is needed to elucidate the deactivating mechanisms of T_1_ state riboflavin by rutin/catechin. In recent years, density functional theory (DFT) calculations have been widely used to study both the photosensitization and deactivation mechanisms of excited state photosensitizers [4, 9–12]. Therefore, in the present study, we attempt to explore how T_1_ state riboflavin was deactivated by rutin/catechin by means of theoretical calculations.

## 2. Theoretical Methods

The calculation procedures are as follows. First, the geometries of riboflavin, rutin and catechin were fully optimized by DFT [14, 15] and B3LYP functional [16–18] with 6–31G(d,p) Gaussian basis set *in vacuo*. Then, the lowest T_1_ excitation energies (E_T1_) of the three molecules were estimated by time-dependent DFT (TD-DFT) [19–21] with the same basis set. Moreover, in view of the fact that the diffusion functions are crucial for treatment of anion and cation radicals, the vertical electron affinities (VEA) and vertical ionization potentials (VIP) of riboflavin, rutin and catechin were calculated by using a combined DFT method labeled as B3LYP/6–31+G(d,p)/B3LYP/6–31G(d,p), which means that B3LYP/6–31+G(d,p) was used to perform a single-point calculation using B3LYP/6–31G(d,p)-optimized geometries [10]. The O-H bond dissociation enthalpy (BDE) of rutin/catechin and H-atom affinity (HAA) of riboflavin were obtained by a hybrid method combining DFT and semiempirical method AM1, labeled as (RO)B3LYP/6–311+G(2d,2p)/AM1, which takes advantages of accuracy and economy [8]. The solvent (benzene and water) effects were taken into account by employing the self-consistent reaction field (SCRF) method with polarizable continuum model (PCM) [22–24] for the single point calculations. All the calculations were accomplished using the Gaussian 03 package of programs [25].

## 3. Results and Discussion

As we know, the ground state photosensitizer is initially excited to the singlet excited state upon irradiation and then may intersystem cross to the relatively long-lived T_1_ state. T_1_ state riboflavin can react with molecular oxygen to photogenerate various ROS [1, 3, 4] and at the same time, it can be deactivated by antioxidants through the following possible pathways:

The first deactivating pathway may proceed through the direct energy transfer between T_1_ state riboflavin (RF) and polyphenols (PhOH) (Equation 1).

(1) $$\text{RF}({\text{T}}_{1}\text{)}\;+\;\text{PhOH}\to \text{RF}+\text{PhOH}({\text{T}}_{1}\text{)}$$

The second deactivating pathway involves the electron transfer between T_1_ state riboflavin and polyphenols (Equation 2).

(2) $$\text{RF}({\text{T}}_{1}\text{)}\;+\;\text{PhOH}\to {\text{RF}}^{\text{.}-}+{\text{PhOH}}^{\text{.}+}$$

Moreover, as the polyphenolic antioxidants are ideal H-atom donors [8], T_1_ state riboflavin may be deactivated by polyphenols through a H-atom transfer process (Equation 3).

(3) $$\text{RF}({\text{T}}_{1}\text{)}\;+\;\text{PhOH}\to {\text{RFH}}^{\text{.}}+{\text{PhO}}^{\text{.}}$$

Therefore, the corresponding electronic parameters of riboflavin, rutin and catechin, including E_T1_, VEA, VIP, O-H BDE and HAA, were estimated and listed in Table 1, according to which, the deactivating reactions of T_1_ state riboflavin by rutin/catechin were analyzed.

Primarily, the E_T1_ of riboflavin, rutin and catechin have been calculated using TD-DFT methods, whose accuracy in estimating the T_1_ state properties of various photosensitizers has been verified [4, 9–13]. It can be seen that the theoretical E_T1_ of rutin/catechin is much higher than that of riboflavin (Table 1), implying that the direct energy transfer-based deactivating pathway (Equation 1) is not feasible on thermodynamic grounds in both solvents.

As to the direct electron transfer-based deactivating pathway (Equation 2), its feasibility depends on the VEA of T_1_ state riboflavin (VEA_T1_) and VIP of rutin/catechin. According to the theoretical results, the summation of VEA_T1_ of riboflavin and VIP of rutin/catechin is positive both in benzene and water, implying that the electron transfer-based deactivating pathway is also not favorable from the thermodynamic point of view.

**Figure 2.**
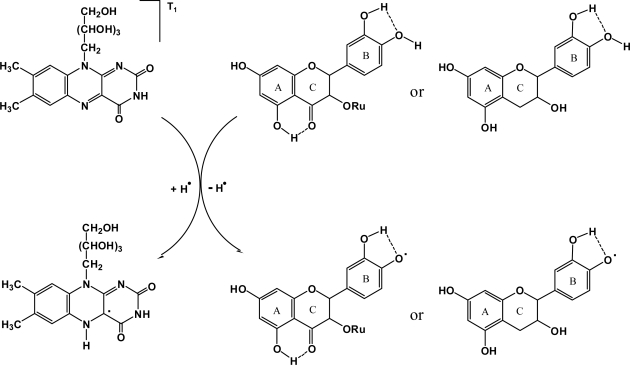
Theoretically postulated H-atom transfer-based triplet excited state riboflavin deactivating pathway by rutin/catechin.

Thirdly, rutin and catechin are excellent H-atom donating substrates [8]. To explore whether the H-atom transfer reaction from rutin/catechin to T_1_ state riboflavin (Equation 3) can occur or not, the O-H BDE of rutin and catechin, which has been successfully used to measure the molecular H-atom-donating ability [8], and the HAA of riboflavin, an appropriate theoretical parameter to characterize the molecular H-atom-abstracting ability [8], have been calculated. Despite the fact that rutin and catechin possess several phenolic hydroxyls that may donate H-atoms, previous studies demonstrated that the hydroxyl at position 4’ (Figure 1) is the most active one [26] and the corresponding O-H BDE in benzene and water is listed in Table 2. The theoretically estimated HAA of T_1_ state riboflavin at N1 (Figure 1), which has been reported to be the thermodynamically favorable position to accept a H-atom [27], is –97.24 kcal/mol in benzene and –106.19 kcal/mol in water (Table 2). As the summation of HAA_T1_ of riboflavin and the O-H BDE of rutin/catechin is negative in both solvents, the H-atom transfer-based quenching pathway is thermodynamically feasible. Therefore, the H-atom transfer-based T_1_ state riboflavin deactivating mechanism by rutin/catechin is proposed as illustrated in Figure 2.

## 4. Conclusions

In summary, through comparing the electronic parameters of riboflavin, rutin and catechin, including E_T1_, VEA, VIP, BDE and HAA, it can be inferred that the H-atom transfer pathway is more feasible on thermodynamic grounds relative to the direct energy transfer or direct electron transfer pathways responsible for the T_1_ state riboflavin deactivation by rutin/catechin. The results have important implications to design/screen better polyphenolic antioxidants as protectors against the photo-oxidative damage induced by riboflavin.

## Figures and Tables

**Table 1.** Theoretically estimated lowest triplet excitation energy (E_T1_, in eV), vertical electron affinity (VEA, in eV) and vertical ionization potential (VIP, in eV) of polyphenols (rutin and catechin) and riboflavin in benzene and water.

Compounds	Solvents	E_T1_	VIP_S0_	VEA_S0_	VEA_T1_^a^
**Rutin**	benzene	3.12	6.76		
	water	3.13	5.95		
**Catechin**	benzene	3.62	6.49		
	water	3.63	5.82		
**Riboflavinb**	benzene	2.10		–2.52	–4.62
	water	2.09		–3.32	–5.41

^a^VEA_T1_ = VEA_S0_ – E_T1_;

^b^data from ref [4].

**Table 2.** Theoretically estimated O-H bond dissociation enthalpy (BDE, in kcal/mol) of the phenolic compounds (rutin and catechin) and T_1_ sate H-atom affinity (HAA_T1_, in kcal/mol) of riboflavin in benzene and water.

Compounds	Solvents	O-H BDE	HAA_T1_^a^
**Rutin**	benzene	78.18	
	water	79.97	
**Catechin**	benzene	78.97	
	water	80.73	
**Riboflavin**	benzene		−97.24
	water		−106.19

^a^HAA_T1_ = HAA_S0_ + E_T1_ .

## References

[1] Baier J, Maisch T, Maier M, Engel E, Landthaler M, Bäumler W (2006). Singlet oxygen generation by UVA light exposure of endogenous photosensitizers. Biophys. J..

[2] Wondrak GT, Jacobson MK, Jacobson EL (2006). Endogenous UVA-photosensitizers: Mediators of skin photodamage and novel targets for skin photoprotection. Photochem. Photobiol. Sci.

[3] Grzelak A, Rychlik B, Bartosz G (2001). Light-dependent generation of reactive oxygen species in cell culture media. Free Radic. Biol. Med.

[4] Shen L, Ji HF, Zhang HY (2007). Computational note on the photosensitization mechanisms of riboflavin. J. Mol. Struct. (Theochem).

[5] Ito K, Inoue S, Yamamoto K, Kawanishi S (1993). 8-Hydroxydeoxyguanosine formation at the 5′ site of 5′-GG-3′ sequences in double-stranded DNA by UV radiation with riboflavin. J. Biol. Chem..

[6] Joshi PC (1985). Comparison of the DNA-damaging property of photosensitised riboflavin via singlet oxygen (^1^O_2_) and superoxide radical O^2-^. mechanisms. Toxicol. Lett.

[7] Becker EM, Cardoso DR, Skibsted LH (2005). Deactivation of riboflavin triplet-excited state by phenolic antioxidants: mechanism behind protective effects in photooxidation of milk-based beverages. Eur. Food. Res. Technol..

[8] Zhang HY (2005). Structure-activity relationships and rational design strategies for radical-scavenging antioxidants. Curr. Computer-Aided Drug Des..

[9] Shen L, Ji HF, Zhang HY (2006). A theoretical elucidation on the solvent-dependent photosensitive behaviors of C_60_. Photochem. Photobiol.

[10] Shen L, Ji HF, Zhang HY (2005). A TD-DFT study on triplet excited-state properties of curcumin and its implications in elucidating the photosensitizing mechanisms of the pigment. Chem. Phys. Lett..

[11] Shen L, Ji HF, Zhang HY (2006). Hypericin anion is crucial to elucidating the pigment’s photosensitive features. Bioorg. Med. Chem. Lett.

[12] Shen L, Ji HF (2008). How α-tocopherol quenches triplet state riboflavin? Insights from theory. J. Photochem. Photobiol. A: Chem..

[13] Shen L, Ji HF (2008). A theoretical study on the quenching mechanisms of triplet state riboflavin by tryptophan and tyrosine. J. Photochem. Photobiol. B: Biol..

[14] Hohenberg P, Kohn W (1964). Inhomogeneous electron gas. Phys. Rev.

[15] Kohn W, Sham LJ (1965). Self-consistent equations including exchange and correlation effects. Phys. Rev.

[16] Lee C, Yang W, Parr RG (1988). Development of the Colle-Salvetti correlation energy formula into a functional of the electron density. Phys. Rev. B..

[17] Becke AD (1993). A new mixing of Hartree-Fock and local density-functional theories. J. Chem. Phys..

[18] Stephens PJ, Devlin FJ, Chabalowski CF, Frisch MJ (1994). Ab Initio calculation of vibrational absorption and circular dichroism spectra using density functional force fields. J. Phys. Chem..

[19] Stratmann RE, Scuseria GE, Frisch MJ (1998). An efficient implementation of time-dependent density-functional theory for the calculation of excitation energies of large molecules. J. Chem. Phys..

[20] Bauernschmitt R, Ahlrichs R (1996). Treatment of electronic excitations within the adiabatic approximation of time dependent density functional theory. Chem. Phys. Lett.

[21] Casida ME, Jamorski C, Casida KC, Salahub DR (1998). Molecular excitation energies to high-lying bound states from time-dependent density-functional response theory: Characterization and correction of the time-dependent local density approximation ionization threshold. J. Chem. Phys..

[22] Miertus S, Scrocco E, Tomasi J (1981). Electrostatic interaction of a solute with a continuum. A direct utilization of *ab initio* molecular potentials for the prevision of solvent effects. Chem. Phys..

[23] Miertus S, Tomasi J (1982). Approximate evaluations of the electrostatic free energy and internal energy changes in solution processes. Chem. Phys.

[24] Cossi M, Barone V, Cammi J (1996). Ab initio study of solvated molecules: a new implementation of the polarizable continuum model. Chem. Phys. Lett..

[25] Frisch MJ, Trucks GW, Schlegel HB, Scuseria GE, Robb MA, Cheeseman JR, Montgomery JA, Vreven T, Kudin KN, Burant JC (2003). Gaussian 03.

[26] Pannala SA, Chan TS, O′Brien PJ, Rice-Evans CA (2001). Flavonoid B-ring chemistry and antioxidant activity: fast reaction kinetics. Biochem. Biophys. Res. Commun..

[27] Cardoso DR, Olsen K, Skibsted LH (2007). Mechanism of deactivation of triplet-excited riboflavin by ascorbate, carotenoids, and tocopherols in homogeneous and heterogeneous aqueous food model systems. J. Agric. Food Chem..

